# Supplementary material for: Movi 2: Fast and Space-Efficient Queries on Pangenomes
Source: bioRxiv. 2025 Oct 30:2025.10.16.682873. Preprint. [Version 2] doi: 10.1101/2025.10.16.682873 (PMC12632922; doi:10.1101/2025.10.16.682873)
Supplement: 1 [file NIHPP2025.10.16.682873v2-supplement-1.pdf]

bioRxiv preprint doi: https://doi.org/10.1101/2025.10.16.682873; this version posted October 30, 2025. The copyright holder for this preprint (which was not certified by peer review) is the author/funder, who has granted bioRxiv a license to display the preprint in perpetuity. It is made available under aCC-BY 4.0 International license.

Supplementary Materials for Movi 2

1. Storing only the non-trivial thresholds

As an advanced version of Movi 1, we implemented a specific thresholds compression approach to store a threshold status instead of an offset for each character. A threshold-offset for each character requires $O(\log_2(\ell_{max}))$ bits (2 bytes in Movi), whereas a threshold-status requires only 2 bits. This is because the status needs to express three main possibilities (assuming the length of the move row is ℓ):

1) The threshold is at the start of the move row (0).
2) The threshold is at the end of the move row (ℓ).
3) a non-trivial offset inside the move row (between 0 and ℓ).

The first two cases do not require any additional information to retrieve the threshold offset. In case (1), the threshold offset is 0 and in case (2), it is set to the length of the the move row. For case (3), the explicit offset of the non-trivial threshold is stored in a dedicated field which requires $O(\log_2(\ell_{max}))$. However, this is the only column requiring $O(\log_2(\ell_{max}))$ bits, which is an improvement over storing $\Sigma - 1$ columns with the same number of bits. It is also possible, though rare in our experiments, for multiple non-trivial thresholds to exist in a row. This can be identified using the 4th code of the threshold status. To handle such cases, we store an overflow table that explicitly stores the thresholds for rows with more than one non-trivial threshold. In practice, we find that this table is small compared to the main Movi table. This approach was implemented in a later release of Movi 1, reducing the total size of each move row from 16 bytes to 12 bytes.

When a trivial threshold is represented by a threshold status rather than full offset, the most significant part of the threshold data structure in Movi becomes the non-trivial thresholds.

2. The blocked index

b = 8

	c	ℓ	f	Δ_ξ	...
1	T	**2**	7	0	...
2	$	**1**	0	0	...
3	T	**2**	0	2	...
4	G	**1**	4	0	...
5	A	**4**	1	0	...
6	C	**2**	0	0	...
7	A	**1**	0	3	...
8	A	**7**	0	0	...
9	C	**1**	2	0	...
...					

The B table (b = 8):

	ξ_A	ξ_C	ξ_G	ξ_T
row 1	1	10	10	11
row 9	8	10	10	-

Fig. 1: The blocked design of Movi 2 where the exact *id* at checkpoints are stored explicitly for each block in the B table. The block size is 8 in this example. The difference from the checkpoint is stored in the move row.

bioRxiv preprint doi: https://doi.org/10.1101/2025.10.16.682873; this version posted October 30, 2025. The copyright holder for this preprint (which was not certified by peer review) is the author/funder, who has granted bioRxiv a license to display the preprint in perpetuity. It is made available under aCC-BY 4.0 International license.

3. Computing the id in the sampled-id mode

Algorithm 1 Computing the id for move row q using the S table. M is the Move table, s is the sampling rate.

Require: M, q, s, S

$C \leftarrow M[q].c$
if $q \ \% \ s = 0$ **then**
  $\xi \leftarrow S[q/s][C]$ ▷ q is at a checkpoint, simply retrieve the sampled id.
else
  $i \leftarrow \lfloor q/s \rfloor \times s$ ▷ the index of the checkpoint in the move table
  $d \leftarrow 0$
  while $q > i$ **do** ▷ step (1) scan and count
    $i \leftarrow i - 1$
    if $M[q].c = C$ **then**
      $d \leftarrow d + M[q].\ell$
    end if
  end while
  $u \leftarrow \lfloor q/s \rfloor$ ▷ step (2) lookup the index of the sampled row in the S table
  $\xi \leftarrow S[u][C]$ ▷ step (3) retrieve the sampled id
  $\ell' \leftarrow M[\xi].\ell - M[i].f$ ▷ the distance from offset until the end of the row
  $d \leftarrow d - \ell'$ ▷ step (4) skip
  while $M[\xi].\ell < d$ **do**
    $\xi \leftarrow \xi + 1$
    $d \leftarrow d - M[\xi].\ell$
  end while
end if
return ξ
